# Color Demosaicing of RGBW Color Filter Array Based on Laplacian Pyramid

**DOI:** 10.3390/s22082981

**Published:** 2022-04-13

**Authors:** Kyeonghoon Jeong, Jonghyun Kim, Moon Gi Kang

**Affiliations:** School of Electrical and Electronic Engineering, Yonsei University, Seoul 03722, Korea; qazwxd91@yonsei.ac.kr (K.J.); jhyun15@yonsei.ac.kr (J.K.)

**Keywords:** color demosaicing, color interpolation, Laplacian pyramid, RGBW color filter array (CFA), white-dominant RGBW

## Abstract

In recent years, red, green, blue, and white (RGBW) color filter arrays (CFAs) have been developed to solve the problem of low-light conditions. In this paper, we propose a new color demosaicing algorithm for RGBW CFAs using a Laplacian pyramid. Because the white channel has a high correlation to the red, green, and blue channels, the white channel is interpolated first using each color difference channel. After we estimate the white channel, the red, green, and blue channels are interpolated using the Laplacian pyramid decomposition of the estimated white channel. Our proposed method using Laplacian pyramid restoration works with Canon-RGBW CFAs and any other periodic CFAs. The experimental results demonstrated that the proposed method shows superior performance compared with other conventional methods in terms of the color peak signal-to-noise ratio, structural similarity index measure, and average execution time.

## 1. Introduction

**Figure 1:**
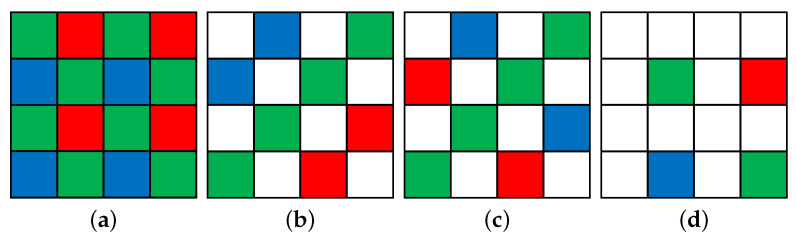
(**a**) Bayer pattern CFA [1], (**b**) Kodak-RGBW CFA [14], (**c**) Sony-RGBW CFA [15], (**d**) Canon-RGBW CFA [16].

Most image acquisition devices use a color filter array (CFA) to acquire color images. Color filter array is structured to obtain color information from a single sensor, which is sampled with each pixel. Each pixel records the value of only one color from the absorbed light. Most commercial cameras use a CFA called the Bayer pattern [1], shown in Figure 1a. The image acquired from the CFA is called the mosaic image, and it requires restoring the missing pixels for each color channel to create a full color image. This is called color demosaicing or color interpolation. It is an essential part of image signal processing.

Since the launch of commercial cameras, numerous color demosaicing algorithms have been developed. Interpolation-based methods such as bilateral and bicubic interpolation appeared in the very early stages of the development. Interpolation-based methods result in unwanted artifacts such as the zipper effect, false color, and blurring. To address these problems, advanced interpolation methods based on high correlation between color channels have emerged. These interpolation methods use the color difference model [2,3]. In addition to the color difference model, several methods for color demosaicing have been developed, such as those based on regularization [4], frequency analysis [5,6], compressive sensing [7,8], and residual interpolation [9,10] using guided filtering [11]. With the recent advances in computer science and equipment, methods have been proposed for interpolating the missing color channels with graphics processing units (GPUs) using convolutional neural networks (CNNs) [12,13]. Most complex demosaicing methods restore the image details, but with high computational costs. Thus, they cannot be applied to industrial products. Therefore, it is important to develop both real-time processing and restoration details.

Autonomous driving technology has developed along with the use of various image acquisition devices. The acquired images help make important judgments in autonomous driving, such as distance measurement, driving speed, and the direction of the vehicle. The image acquisition device used for autonomous driving may be a LiDAR or a general camera. LiDAR accurately measures distance. However, due to the high price and low resolution of LiDAR, several commercial cameras are used in autonomous vehicles.

Despite advances in color demosaicing algorithms, the problem of low-light conditions remains. Because autonomous driving also accounts for a large part of night driving, images in low-light conditions are important. In low-light conditions, noise is noticeable because the signal’s power is reduced. To solve this problem, several methods have been developed. One of the methods includes increasing the sensor’s size. However, the size of the sensor of commercial cameras cannot be increased sufficiently due to spatial constraints. Another method requires the camera user to increase the exposure time; however, if the exposure time is increased, motion blurring artifacts may occur. Changing the pattern of the CFA to improve image quality in low-light conditions is another method. Numerous CFA patterns containing a white channel that receives the full range of visible light have been designed, such as the Kodak-RGBW CFA, shown in Figure 1b [14], the Sony-RGBW CFA, shown in Figure 1c [15], and the Canon-RGBW CFA, shown in Figure 1d [16]. These patterns solve the problem of image quality in low-light conditions because the spectral sensitivity of white is higher than that of red, green, and blue. The greater the number of white pixels in the CFA, the more resistant the CFA is to noise in low-light conditions.

The contributions of this paper are summarized as follows. We propose an optimized color demosaicing algorithm for the Canon-RGBW CFA. Moreover, white channel interpolation based on the gradient of color difference models is proposed. We then propose color channel demosaicing using a Laplacian pyramid based on the structural similarity of the subsampled color channels. We compare the existing state-of-the-art (SOTA) methods to our proposed method with real data, Kodak, and the McMaster datasets [17] using noise-free and high noisy images. Our method achieved higher image quality than the SOTA methods based on metrics such as PSNR and SSIM. Our method is also applicable to other periodic CFAs.

## 2. Related Work

### 2.1. Frequency Analysis of CFAs

In this study, it was necessary to select an appropriate CFA that contains white for good performance in normal-light and low-light conditions. If the ratio of white is very high in a CFA, it is robust against extreme low-light conditions, but there is a problem with color bleeding. Therefore, we needed a CFA with an appropriate ratio of white. Hao et al. [18] proposed analyzing various CFAs through a combination of luminance and chrominance in the frequency domain. Through Hao’s method [18], we explain the reason for selecting the Canon-RGBW CFA. The paper [18] proposed several principles for a good CFA. One is to design the distance between luminance and chrominance to be as long as possible in the frequency domain. The second is to design many repeated chrominances. The former principle is for accurate luminance estimation, and the latter is for accurate color estimation. The two conditions have a trade-off relationship with one other.

**Figure 2:**
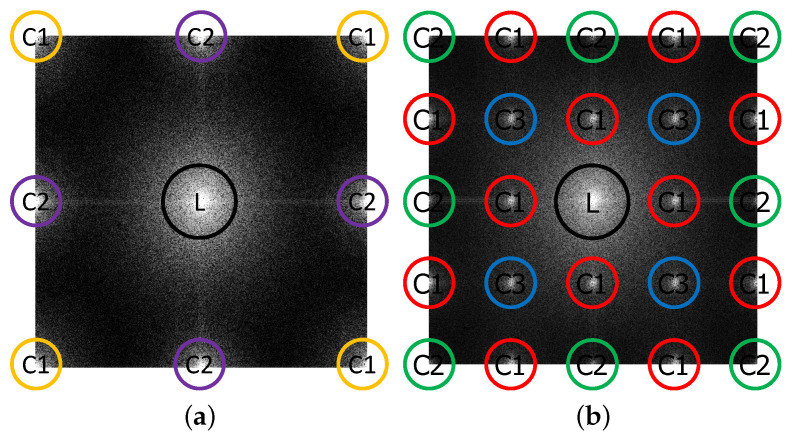
(**a**) Frequency analysis of Bayer pattern CFA, (**b**) Frequency analysis of Canon-RGBW CFA.

Figure 2a shows the frequency analysis of the McMaster16 dataset for the Bayer pattern CFA, and Figure 2b shows the frequency analysis of the McMaster16 dataset for the Canon-RGBW CFA. L stands for luminance, and C1, C2, and C3 stand for different chrominances in Figure 2. In the case of Figure 2a, the distance between the luminance and chrominance is long, but there are few repeated chrominances. However, Figure 2b shows a short distance between the luminance and chrominance, but many repeated chrominances exist. The distance between the luminance and chrominance is short in the frequency domain, but the higher percentage of white in the spatial domain of Canon-RGBW CFA allows for a more accurate estimation of the white channel. Because the white channel has characteristics similar to luminance, the disadvantage can be offset. Furthermore, the Canon-RGBW CFA has a simple sampling period for color channels, such as that corresponding to an integer multiple of the Bayer pattern CFA. It is an appropriate array to utilize with the Laplacian pyramid [19].

### 2.2. Traditional Methods of Color Demosaicing

Most conventional color demosaicing methods have been developed using color difference or residual domain. Both methods are performed in two steps for the Bayer pattern CFA. The first step is interpolating the green channel with the maximum color information. Then, other colors are interpolated using the interpolated green channel.

The color difference domain is based on the idea that red and blue colors are highly correlated with the green color. We proceed with demosaicing using the difference between green and another color. Interpolation is required for each color channel because all values are not present. For example, green pixels are half, and the red and blue colors each occupy a quarter of the image size in the Bayer pattern CFA. To interpolate each color channel with no pixel values, either the Hamilton and Adams’ [20] interpolation formula or a bilinear filter is used. The color difference domain is calculated using previously interpolated color channels, as follows:

(1) $$\begin{array}{cc} {\tilde{\Delta }}_{G-R}= & \tilde{G}-\tilde{R},\\ {\tilde{\Delta }}_{G-B}= & \tilde{G}-\tilde{B}, \end{array}$$

where $\tilde{G}$ and $\tilde{R}$ are fully interpolated color channels and $\tilde{\Delta }$ is the color difference. The color difference $\tilde{\Delta }$ is estimated using bilinear or edge directional interpolation. Pei et al. proposed a method for generating color difference domains using bilinear interpolation [21] and Pekkucuksen et al. proposed a method for generating color difference domains by discriminating four directions [22]. The estimated color differences are added to the original pixel values to estimate the final green channel:

(2) $$\begin{array}{c} G=\left\{\begin{array}{c} {\tilde{\Delta }}_{G-R}+R_{CFA}\;\mathrm{at}\;\mathrm{red}\;\mathrm{pixels},\\ {\tilde{\Delta }}_{G-B}+B_{CFA}\;\mathrm{at}\;\mathrm{blue}\;\mathrm{pixels}, \end{array}\right. \end{array}$$

Kiku et al. developed the gradient-based-threshold-free (GBTF) method and proposed a residual domain [9]. After estimating the green channel using the GBTF method, it was used as a guide image. They obtained temporary red and blue channels using guided images, as follows:

(3) $$\begin{array}{cc} R_{tent}= & GF(G,R_{CFA}),\\ B_{tent}= & GF(G,B_{CFA}), \end{array}$$

where tent means tentative, $GF(input_{A},input_{B})$ means guided filtering, $input_{A}$ means the guide image, and $input_{B}$ means the filtering input image. They defined the difference between the original CFA and the tentative color image as the residual image.

(4) $$\begin{array}{cc} {\tilde{R}}_{resi}= & R_{CFA}-R_{tent}\;\mathrm{at}\;\mathrm{red}\;\mathrm{pixels},\\ {\tilde{B}}_{resi}= & B_{CFA}-B_{tent}\;\mathrm{at}\;\mathrm{blue}\;\mathrm{pixels}. \end{array}$$

Because ${\tilde{R}}_{resi}$ and ${\tilde{B}}_{resi}$ are masked images, we need to interpolate them to generate fully residual images ${\hat{R}}_{resi}$ and ${\hat{B}}_{resi}$. The estimated color images *R* and *B* are calculated by the summation of residual and tentative images, as follows:

(5) $$\begin{array}{cc} R= & {\hat{R}}_{resi}+R_{tent},\\ B= & {\hat{B}}_{resi}+B_{tent}. \end{array}$$

The demosaiced output generated using the residual domain is better than that generated using the color difference domain. However, the computational cost is high.

### 2.3. Laplacian Pyramid

**Figure 3:**
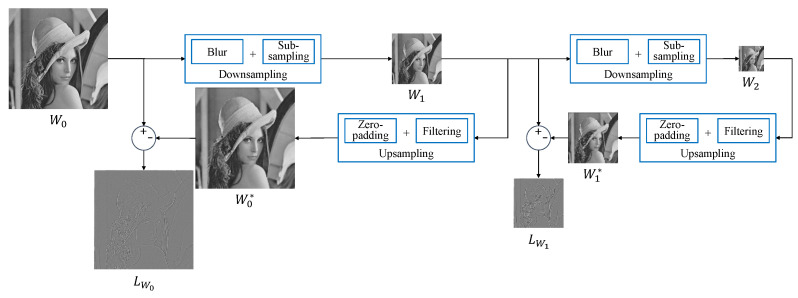
Outline of Laplacian pyramid decomposition with depth level l=2.

A Laplacian pyramid consists of downsampled images and high-frequency components of the image. We call the high-frequency components of the image detailed images. Because only one downsampled image and detailed image of each level are needed to restore the original, a Laplacian pyramid is commonly used for image compression and detail enhancement [23]. The detailed image is the differences between the blurred image and its previous downsampled image at each level. For example, the blurred image $W_{0}^{*}$ is the upsampled image of $W_{1}$, and the image $W_{1}$ is the downsampled image of $W_{0}$. The detailed image $L_{0}$ is the difference between $W_{0}$ and the blurred image $W_{0}^{*}$. Figure 3 shows the flowchart of a Laplacian pyramid decomposition with depth level l=2.

The downsampled image of the next level is computed in the order of subsampling after Gaussian blurring:

(6) $$\begin{array}{cc} W_{l+1}\left(i,j\right)= & \sum_{x}\sum_{y}g\left(x,y\right)W_{l}\left(2i-x+m,2j-y+n\right)\;\;\left(m,n\right)\in {\left[0,1\right]}^{2} \end{array}$$

where *l* is the index of the depth level, g(x,y) is a Gaussian kernel that creates blur, and (m,n) is the location value that indicates where the image is subsampled. When we create a detailed image $L_{l}$, we first perform upsampling on the downsampled image from the previous level as below:

(7) $$W_{l}^{\prime }\left(2i+m,2j+n\right)=W_{l+1}\left(i,j\right).$$

We need two steps to upsample the downsampled image. The first step is to create an image that doubles the downsampled image. At this time, the value is filled at the subsampled position (m,n) and the remainder is filled with 0. This step is regarded as zero padding. The second step is to create a final upsampled image through low-pass filtering of the zero-padded image. Therefore, ref. [7] means zero padding to double the image size. After zero padding, it is necessary to filter for complete upsampling, as shown below:

(8) $$W_{l}^{*}\left(i,j\right)=\sum_{x}\sum_{y}h\left(x,y\right)W_{l}^{\prime }\left(i-x,j-y\right),$$

where h(x,y) is the interpolation filter, such as the bilinear filter. The detailed image of the *l*th level is the differences between $W_{l}$ and the blurred image $W_{l}^{*}$,

(9) $$L_{W_{l}}\left(i,j\right)=W_{l}\left(i,j\right)-W_{l}^{*}\left(i,j\right).$$

**Figure 4:**
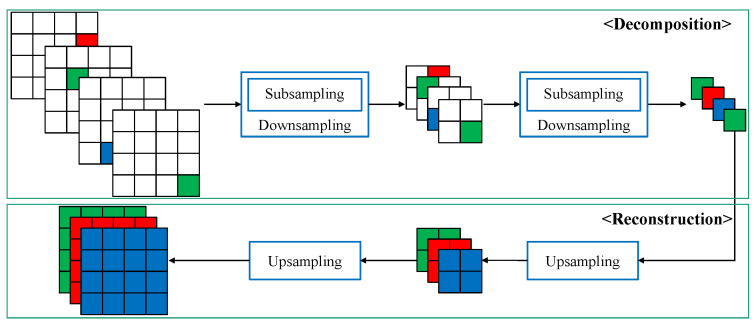
Structural similarity of subsampled Canon-RGBW CFA and Laplacian pyramid.

As we can see in Figure 4, the subsampled color channels of the Canon-RGBW CFA have a structure similar to the Laplacian pyramid in Figure 3. If we sub-sample the Canon-RGBW CFA twice to create each color channel, we observe that it is structurally similar to a Laplacian pyramid decomposition with depth level l=2. The only difference is that here there is no Gaussian filtering in downsampling. This means that g(x,y) is a Dirac delta function in (6). If we can estimate the detailed images of each color channel, such as the detailed images $L_{W_{0}}$, $L_{W_{1}}$ in Figure 3, we can restore the full resolution color image. This is similar to reconstruction in the Laplacian pyramid.

## 3. Proposed Algorithm

This section describes our proposed algorithm, which comprises two steps. First, we interpolate the white channel using the color difference model. Second, we interpolate the red, green, and blue channels using Laplacian pyramid reconstruction.

### 3.1. White Channel Interpolation

Most demosaicing methods designed for the Bayer pattern CFA begin by interpolating the green channel, whereas our algorithm starts by interpolating the white channel. This is because the white channel has high sensitivity, is noise-resistant, and performs good in low-light conditions. Moreover, the white channel has a higher spatial resolution than the other channels. The Canon-RGBW CFA consists of three times as many white pixels as pixels for the other colors, as depicted in Figure 1d. Therefore, the white channel is advantageous for edge direction determination. Third, the white channel is highly correlated with the red, green, and blue color channels. This is an important key to restoring the missing color pixel values.

We utilize a gradient-based-threshold-free (GBTF) algorithm [22] for the Canon-RGBW CFA. The GBTF algorithm is designed for the Bayer pattern CFA. Therefore, we modify the GBTF algorithm to apply to the Canon-RGBW CFA. First, we perform the interpolation for the white and color pixel values in the vertical and horizontal directions. The directional estimates for the missing white and color pixel values are calculated as follows:

(10) $$\begin{array}{cc} {\tilde{W}}^{H}\left(i,j\right) & =\left\{\begin{array}{cc} W\left(i,j-1:j+1\right)\cdot f_{1}^{T} & \mathrm{at}\;\mathrm{color}\;\mathrm{pixels}\\ W(i,j) & \mathrm{at}\;\mathrm{white}\;\mathrm{pixels}, \end{array}\right.\\ {\tilde{C}}^{H}\left(i,j\right) & =C\left(i,j-3:j+3\right)\cdot f_{2}^{T}\\ {\tilde{W}}^{V}\left(i,j\right) & =\left\{\begin{array}{cc} W\left(i-1:i+1,j\right)\cdot f_{1} & \mathrm{at}\;\mathrm{color}\;\mathrm{pixels}\\ W(i,j) & \mathrm{at}\;\mathrm{white}\;\mathrm{pixels}, \end{array}\right.\\ {\tilde{C}}^{V}\left(i,j\right) & =f_{2}\cdot C(i-3:i+3,j),\\ f_{1} & =[\frac{1}{2}\;0\;\frac{1}{2}],\\ f_{2} & =[\frac{1}{4}\;\frac{1}{2}\;\frac{3}{4}\;1\;\frac{3}{4}\;\frac{1}{2}\;\frac{1}{4}], \end{array}$$

where *W* and *C* denote the white channel and the color channel, e.g., red, green, and blue channels, and *H* and *V* denote horizontal and vertical directions. Equation (10) can also be viewed as a bilinear filter for directional interpolation. The next step is to calculate the horizontal and vertical color differences:

(11) $$\begin{array}{cc} {\tilde{\Delta }}_{W,C}^{H}\left(i,j\right) & ={\tilde{W}}^{H}\left(i,j\right)-{\tilde{C}}^{H}\left(i,j\right)\\ {\tilde{\Delta }}_{W,C}^{V}\left(i,j\right) & ={\tilde{W}}^{V}\left(i,j\right)-{\tilde{C}}^{V}\left(i,j\right). \end{array}$$

The absolute gradients of the color difference are defined as follows:

(12) $$\begin{array}{cc} D_{W,C}^{H}\left(i,j\right)= & \left|{\tilde{\Delta }}_{W,C}^{H}\left(i,j-1\right)-{\tilde{\Delta }}_{W,C}^{H}\left(i,j+1\right)\right|\\ D_{W,C}^{V}\left(i,j\right)= & \left|{\tilde{\Delta }}_{W,C}^{V}\left(i-1,j\right)-{\tilde{\Delta }}_{W,C}^{V}\left(i+1,j\right)\right|. \end{array}$$

We use the absolute gradients of color difference in (12) for the edge weight calculation. For white channel interpolation, we estimate the directional color difference in (11), and then we combine them accordingly:

(13) $$\begin{array}{cc} {\hat{\Delta }}_{W,C}\left(i,j\right)= & [w_{H}\cdot {\tilde{\Delta }}_{W,C}^{H}\left(i,j-4:j+4\right)\cdot f_{3}^{T}\\ \; & +w_{V}\cdot f_{3}\cdot {\tilde{\Delta }}_{W,C}^{V}\left(i-4:i+4,j\right)]/w_{C}\\ f_{3}= & [\frac{1}{4}\;0\;0\;0\;\frac{1}{2}\;0\;0\;0\;\frac{1}{4}]. \end{array}$$

For a local window size of 9×9, the weights for the vertical and horizontal directions are calculated as follows:

(14) $$\begin{array}{cc} w_{H} & =1/{\left(\sum_{k=i-4}^{i+4}\sum_{l=j-4}^{j+4}D_{W,C}^{H}\left(k,l\right)\right)}^{2}\\ w_{V} & =1/{\left(\sum_{k=i-4}^{i+4}\sum_{l=j-4}^{j+4}D_{W,C}^{V}\left(k,l\right)\right)}^{2}\\ w_{C} & =w_{H}+w_{V}. \end{array}$$

Equation (13) describes the directional adaptive low-pass filtering of the differences between color channels, because the color difference model assumes that the differences between color channels vary slowly. Finally, it is possible to refine the estimated value of a pixel by updating the initial color difference estimates using its four neighbor weights. The final color difference can be given as follows:

(15) $$\begin{array}{cc} {\tilde{\Delta }}_{W,C} & \left(i,j\right)=[w_{N}\cdot {\hat{\Delta }}_{W,C}\left(i-4,j\right)+w_{S}\cdot {\hat{\Delta }}_{W,C}\left(i+4,j\right)\\ \; & +w_{W}\cdot {\hat{\Delta }}_{W,C}\left(i,j-4\right)+w_{E}\cdot {\hat{\Delta }}_{W,C}\left(i,j+4\right)]/w_{T}. \end{array}$$

(16) $$\begin{array}{cc} w_{N} & =1/{\left(\sum_{k=i-4}^{i}\sum_{l=j-4}^{j+4}D_{W,C}^{V}\left(k,l\right)\right)}^{2}\\ w_{S} & =1/{\left(\sum_{k=i}^{i+4}\sum_{l=j-4}^{j+4}D_{W,C}^{V}\left(k,l\right)\right)}^{2}\\ w_{W} & =1/{\left(\sum_{k=i-4}^{i+4}\sum_{l=j-4}^{j}D_{W,C}^{H}\left(k,l\right)\right)}^{2}\\ w_{E} & =1/{\left(\sum_{k=i-4}^{i+4}\sum_{l=j}^{j+4}D_{W,C}^{H}\left(k,l\right)\right)}^{2}\\ w_{T} & =w_{N}+w_{S}+w_{W}+w_{E}. \end{array}$$

The weights $\left(w_{N},w_{S},w_{W},w_{E}\right)$ are calculated in (16). The vertical direction weights $\left(w_{N},w_{S}\right)$ use a local window size of 9×5 and the horizontal direction weights $\left(w_{W},w_{E}\right)$ use 5×9 window.

After calculating the final color difference in (15), we add it to the target color pixel value. The final interpolated white channel is interpreted as:

(17) $$\begin{array}{c} W\left(i,j\right)=C\left(i,j\right)+{\tilde{\Delta }}_{W,C}\left(i,j\right)\;\mathrm{at}\;\mathrm{each}\;\mathrm{color}\;\mathrm{pixels}. \end{array}$$

### 3.2. Red, Green, and Blue Channels Interpolation

The color difference model is based on the high correlation between each color channel. In general, a high correlation between two variables does not mean that they are the same. However, the smoothness in the color difference domain implies that the high-frequency components from different color channels are correlated and are similar to each other. The approximate equality of high-frequency components between different color channels is well-known in demosaicing [3,24].

**Figure 5:**
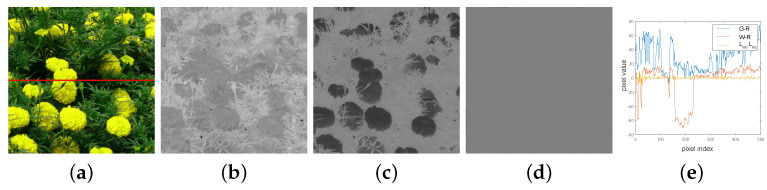
Examples of (**a**) RGB image, (**b**) $G_{0}-R_{0}$, (**c**) $W_{0}-R_{0}$, (**d**) $L_{W_{0}}-L_{R_{0}}$, (**e**) pixel values of the red line location of (**a**) in (**b**–**d**).

The color channel is reconstructed based on Laplacian pyramid reconstruction. We demonstrate that our proposed method is better than conventional methods. Figure 5 shows examples of (a) an RGB image, (b) a color difference image of $G_{0}-R_{0}$, (c) a color difference image of $W_{0}-R_{0}$, (d) a Laplacian difference image of $L_{W_{0}}-L_{R_{0}}$, and (e) the pixel values of the red line’s location in Figure 5a in each of Figure 5b–d. The Laplacian difference in Figure 5d varies more slowly than the color difference in Figure 5b,c. The pixel values of the orange line are smaller than those of the red and blue lines in Figure 5e. The smaller the pixel values in the color difference image, the higher the correlation between each color image. This means that the Laplacian difference model is more advantageous than the color difference model for color interpolation.

The white image *W* and the color image *C* consist of low-frequency components $W^{low}$ and $C^{low}$ and the high-frequency components $W^{high}$ and $C^{high}$, as follows:

(18) $$\begin{array}{cc} W & =W^{low}+W^{high}\\ C & =C^{low}+C^{high}. \end{array}$$

Because $W_{0}^{*}$ is upsampled after the image $W_{0}$ in Figure 3 is downsampled, it can be considered a low-frequency component. Thus, $W^{low}$ in (18) can be regarded as $W_{0}^{*}$. The detailed image in a Laplacian pyramid means that it is a high-frequency component of the original image. Therefore, $W^{high}$ in (18) can be regarded as the detailed image $L_{W_{0}}$. We define the low- and high-frequency components for each depth level *l* as follows:

(19) $$\begin{array}{cc} W_{l}^{1ow}= & W_{l}^{*},\;W_{l}^{high}=L_{W_{l}}\\ C_{l}^{low}= & C_{l}^{*},\;\;\;C_{l}^{high}=L_{C_{l}}. \end{array}$$

We obtain the low-frequency components $W^{low}$ and $C^{low}$ by upsampling the downsampled image in the Laplacian pyramid. We define upsampling in a Laplacian pyramid as a bilinear method for lower computations. Thus we need to perform the Laplacian pyramid decomposition for the estimated white channel. If we interpolate the red channel, we choose the values m=0 and n=1 for the Canon-RGBW CFA, as follows:

(20) $$\begin{array}{cc} W_{2}\left(i,j\right)= & W_{1}\left(2i+m,2j+n\right)\;\;\left(m,n\right)\in {\left[0,1\right]}^{2}. \end{array}$$

**Figure 6:**
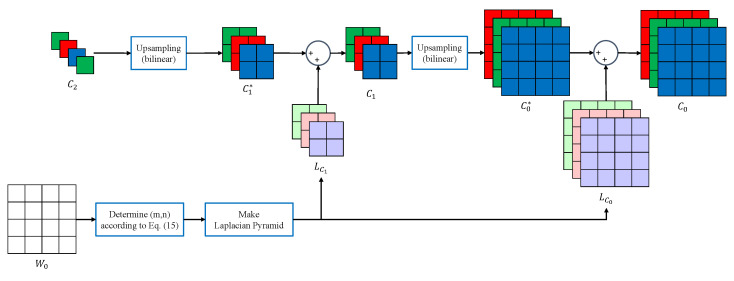
The proposed method for color channel interpolation using Laplacian pyramid reconstruction.

After choosing the value of (m,n), we upsample the image $W_{2}$ to calculate the detailed image $L_{W_{1}}$. We compute the detailed image $L_{W_{0}}$ in the same way as before. To do our proposed color channel interpolation, we need the detailed images $L_{C_{0}}$ and $L_{C_{1}}$ for each color channel. Figure 6 shows the outline of the proposed interpolation for the color channels. The aforementioned assumption is that the high-frequency components shared between the color channels can be approximated as the same. Therefore, we can assume that the high-frequency components of the color channel $L_{C_{0}}$ and $L_{C_{1}}$ are approximately the same as those of the white channel $L_{W_{0}}$ and $L_{W_{1}}$.

After acquiring a detailed image with *W*, the image goes through the process of restoration to a full-resolution image. We summarize the proposed color channel interpolation based on a Laplacian pyramid in Algorithm 1.
**Algorithm 1: **Color Interpolation using the Laplacian Pyramid**   Input**: The estimated image $W_{0}$,      The subsampled CFA $C_{l}$**   Output**: The reconstructed color image $C_{0}$,           C∈R,G,B**_1_** Decide (m,n) according to (20) $\left(m,n\right)=\left\{\begin{array}{cc} (0,1), & \mathrm{at}\;C=R\\ \{(0,0),(1,1)\}, & \mathrm{at}\;C=G\\ (1,0), & \mathrm{at}\;C=B \end{array}\right.$**_2_** Make Laplacian pyramid about *W* with (m,n) and depth level *l***_3_ while**l>0
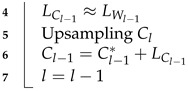
**_8_ return**$C_{0}$

## 4. Experiment Results

**Figure 7:**
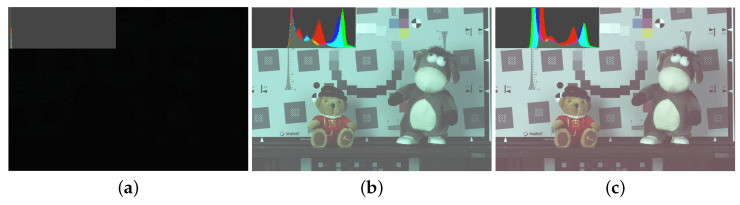
Images and its histograms: (**a**) The acquired image without post processing, (**b**) Linear stretching of (**a**), (**c**) The result with linear stretching and white balancing.

For the experimental evaluation of our proposed algorithm, we compared the qualities of the real captured image based on the Canon-RGBW CFA and the Bayer pattern CFA. Experimental results show that the RGBW CFA is more robust to noise than the Bayer pattern CFA in low-light conditions. Furthermore, spatial information could be obtained from the RGBW CFA results but not Bayer pattern CFA results. To obtain the full-resolution images of the red, green, blue, and white channels, we used a filter wheel camera. The filter wheel camera can be rotated in the clockwise or counterclockwise direction for filter selection, and it has four filters: red, green, blue, and white. We took the full resolution images of the red, green, blue, and white channels of the same scene using the four different filters. The exposure time was set to 1/60 s under 5 lux illumination. Using the full-resolution images of the four channels, we performed sampling to fit the Bayer and Canon-RGBW CFA patterns. The image acquired at 5 lux illumination was very dark, as shown in Figure 7a. We performed linear stretching, which is the simplest method to increase pixel values (Figure 7b). We multiplied pixels that acquired raw data by a large number (in this case, 25) and then performed white balancing, as shown in Figure 7c. We performed the simplest white balancing method, which is the gray world method [25].

**Figure 8:**
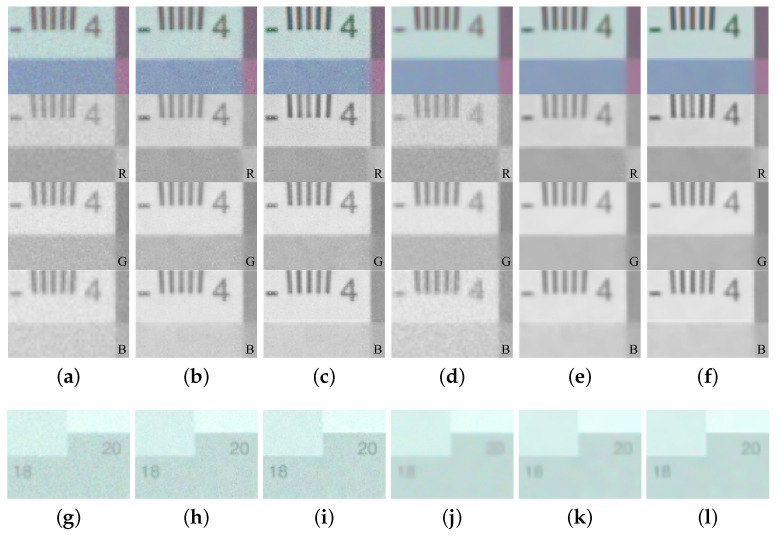
Experimental results with 5 lux low-light condition: (**a**) The demosaiced image using the Bayer pattern CFA with RI [10], (**b**) The demosaiced image using the Canon-RGBW CFA with modified RI [10], (**c**) The demosaiced image using the Canon-RGBW CFA with proposed method, (**d**–**f**) The results applying BM3D on (**a**–**c**). (**g**–**l**) use the same methods as (**a**–**f**).

Figure 8 shows the results of applying the same RI method of the Bayer pattern CFA and the Canon-RGBW CFA pattern and those of applying the proposed method in low-light conditions. Figure 8a,b show that the Canon-RGBW CFA is more robust to noise than the Bayer pattern CFA, according to the results of applying the same RI method to the two CFAs. This means that the CFA containing a white channel is more robust to noise than the Bayer pattern CFA. Figure 8b,c show the results of applying the RI and proposed methods with the same Canon-RGBW CFA. It can be observed that the result of the proposed method exhibits more high frequency information compared to that of the RI method. Furthermore, we compared the results of applying the same denoising method [26], BM3D(Block-matching and 3D filtering), to Figure 8a–c. The BM3D result of the Canon-RGBW CFA contained more image details than that of the Bayer pattern CFA, as shown in Figure 8d,e, and the objects which applied the proposed method are better identified, as shown in Figure 8e,f.

**Figure 9:**
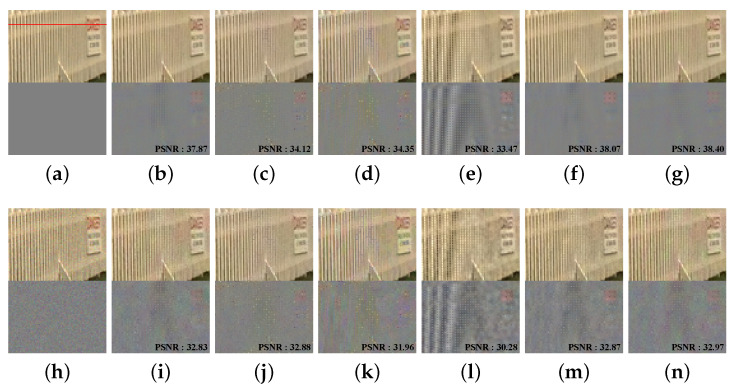
Visual comparison of the color demosaiced image from the noise-free and noisy datasets with $\sigma_{n}=0.03$. The upper parts of each row show the enlarged images, and the lower parts of each row show the difference maps. The first row shows the results of color demoisaicing without noise, and the second row shows noise with $\sigma_{n}=0.03$: (**a**) Ground truth, (**b**) CM1 [10], (**c**) CM2 [27], (**d**) CM3 [28], (**e**) CM4 [13,21], (**f**) CM5 [13], (**g**) PM. (**h**–**n**) use the same methods as (**a**–**g**).

**Figure 10:**
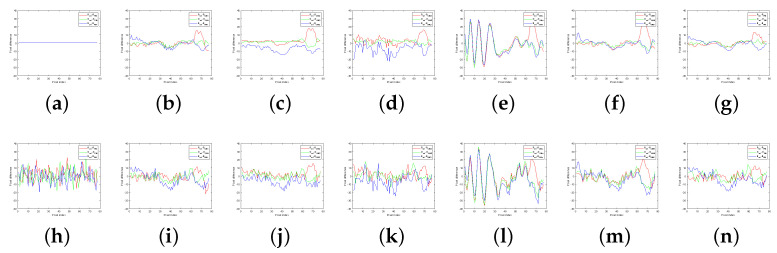
(**a**–**n**) The difference between original and each method in Figure 9a–n at the red line location in Figure 9a.

The qualitative evaluation of the Kodak dataset without noise and with high noise is shown in Figure 9. In the noise-free dataset, CM1 shows good results in terms of color fidelity. However, it can be seen that there is a slight color error in the vertical direction of the fence and in the red letters on the signs. CM2 and CM3 have a color bleed artifact. The difference maps for CM2 and CM3 are brighter than that of the original. This is a typical color loss phenomenon that is called color bleeding. CM2 and CM3 show partially incorrect color estimation, as we can see yellow pixels in the each difference map. CM4 has aliasing in the fence. CM5 shows good results. However, the red letters on the signs also show color bleeding. The proposed method based on the Laplacian pyramid shows results similar to CM5, but the color bleeding is reduced. In other words, the proposed algorithm produces the best visual result. Figure 9h is the image generated by adding Gaussian noise with a standard deviation of $\sigma_{n}=0.03$ to the original image. In the high-noise condition, the noisy dataset shows a similar tendency to the noise free condition. Most of the methods show good results, even in high-noise environments that could be considered low-light conditions. However, CM1 looks somewhat more resistant to noise than the proposed method. CM2 and CM3 still show color bleeding. CM4 obtains the worst results. CM5 is similar to the proposed method. However, it shows more color bleeding in the red letters on the signs. Figure 10 shows the difference between original image and the results of Figure 9 at the red line in Figure 9a. The red, green, and blue lines in Figure 10 are the difference pixel values of the red, green, and blue channel.

In addition to experiments on real captured images, we used the Kodak and McMaster dataset [17] for demosaicing comparison. The Kodak dataset consists of 24 color images of size 768×512 and the McMaster dataset consists of 18 color images of size 500×500. Because the Kodak and McMaster datasets have no original white pixel values, we assume that the values of the white pixels are a summation of the red, green, and blue pixel values at the same location; that is, W=(R+G+B). We conducted experiments on two types of degradation. The synthetic dataset was created without noise and with Gaussian noise having a standard deviation of $\sigma_{n}=0.03$. An image in low-light conditions has low signal power. Its signal-to-noise ratio (SNR) value is small. This means that it is equivalent to an image under normal-light conditions with added noise. It can be assumed that as the standard deviation of the noise increases, the light in the environment decreases. We chose Gaussian noise with a standard deviation of $\sigma_{n}=0.03$ because the average SNR values of the Kodak and McMaster datasets are 15.9 dB and 17.6 dB. It can be assumed to be a low-light condition.

We used two metrics to measure the performance of the proposed method. One was the color peak signal-to-noise ratio (CPSNR) and the other was the structural similarity index (SSIM) [29]. CPSNR is measured using the mean square error of the original image and the estimated image, and SSIM evaluates the similarity of structures and features between the two images as perceived by the human visual system.

The proposed method for performance evaluation was compared with five existing methods. All the conventional methods were implemented using the Canon-RGBW CFA. The first conventional method (CM1) was the RI method [10], which did not use the color difference model but the residual model. The second (CM2) was Oh’s method [27], which contained the colorization-based method. The third (CM3) was Kim’s method [28], which uses rank minimization with a colorization constraint. The fourth (CM4) and fifth (CM5) were demosaicnet [13], based on deep learning. Because there is no learning-based method for the Canon-RGBW CFA, we used a hybrid method combined with the existing color demosaicing method. We implemented deep learning with the Bayer pattern CFA, such as the red, green, and blue pixel locations in the Canon-RGBW CFA. Thereafter, we applied handcrafted methods. In other words, demosaicnet was applied to a Bayer pattern-like image that was subsampled once using the Canon-RGBW CFA. CM4 was combined with demosaicnet [13] and the color difference model [21], and CM5 was combined with demosaicnet [13] and the proposed method based on a Laplacian pyramid.

The quantitative evaluation of the noise-free Kodak dataset in terms of CPSNR and SSIM is presented in Table 1. In Table 1, Table 2 and Table 3, the table colored in vivid sky blue indicates the highest score and the table colored in light sky blue indicates the second highest score. A high CPSNR value means that the original and estimated images are quite similar and a high SSIM value also means that the estimated image is structurally similar to the original image. It can be observed that the proposed method shows improved performance. Of all the methods, including the residual, colorization, rank minimization, and hybrid methods, the proposed method achieved most of the highest CPSNR and SSIM values on the Kodak dataset. We also achieved the best results for the average CPSNR and SSIM. We summarize the average CPSNR and SSIM values for the Kodak and McMaster dataset without noise and containing added noise with $\sigma_{n}=0.03$ in Table 2. For the most part, it can be observed that the proposed method and the hybrid method that combines deep learning with the proposed Laplacian pyramid show good performance.

To evaluate the time complexity of the conventional methods and the proposed method, we estimated their average execution time on the Kodak dataset. The codes of conventional methods are available online. All the codes were written in Matlab files except the demosaicnet and tested on MATLAB R2021a. The demosaicnet model was built in PyTorch 1.8.1 and tested on Ubuntu 16.04 environment (Python3.8, CUDA11.2). We used a desktop computer equipped with an Intel Core i7-11700k 3.6GHz CPU, 32 GB memory, and an Nvidia RTX-3090 GPU. The results are listed in Table 3. Because CM4 and CM5 are deep learning-based hybrid methods, the average execution time is the total time that the CPU and GPU operate. Our proposed method consumes much less time than the conventional methods.

## 5. Conclusions

In this paper, we proposed a color demosaicing method for the Canon-RGBW CFA using a Laplacian pyramid. First, we interpolated the white channel utilizing the color difference model and gradient. Red, green, and blue channels were restored with our proposed method using Laplacian pyramid decomposition and restoration. Our method can be utilized with any periodic CFA. The experimental results showed that the proposed method has superior results compared with the other conventional methods, including quantitative, visual performance, and computational complexity. As can be seen from the experimental results, our method still shows color bleeding with the Canon-RGBW CFA. To alleviate this, we will experiment by adding appropriate regularization.

## Figures and Tables

**Table 1:** Comparison of CPSNR(DB) and SSIM (Noise free) on the Kodak dataset.

Kodak Dataset												
	**CPSNR**						**SSIM**					
**No**.	**CM1**	**CM2**	**CM3**	**CM4**	**CM5**	**PM**	**CM1**	**CM2**	**CM3**	**CM4**	**CM5**	**PM**
1	34.53	32.21	32.23	32.11	36.72	35.40	0.9792	0.9629	0.9634	0.9719	0.9879	0.9890
2	36.71	33.74	35.54	35.95	37.36	37.30	0.9890	0.9855	0.9921	0.9933	0.9946	0.9933
3	37.66	32.26	36.50	38.53	40.40	39.26	0.9911	0.9774	0.9885	0.9913	0.9949	0.9975
4	36.71	33.56	34.79	36.99	38.44	36.98	0.9897	0.9828	0.9891	0.9917	0.9938	0.9961
5	32.34	30.08	32.03	30.78	32.48	32.81	0.9742	0.9572	0.9726	0.9685	0.9781	0.9831
6	35.81	33.01	32.75	32.94	36.97	37.11	0.9841	0.9688	0.9691	0.9763	0.9897	0.9924
7	37.43	34.16	36.42	36.67	38.31	38.18	0.9885	0.9810	0.9893	0.9890	0.9923	0.9958
8	32.74	31.81	32.47	29.05	33.50	34.22	0.9787	0.9661	0.9736	0.9632	0.9827	0.9878
9	39.23	35.80	38.06	37.15	40.38	39.79	0.9855	0.9710	0.9770	0.9774	0.9874	0.9921
10	38.23	36.46	37.83	35.87	37.45	39.43	0.9844	0.9705	0.9785	0.9773	0.9857	0.9905
11	36.18	33.01	33.70	33.40	36.05	36.72	0.9830	0.9637	0.9720	0.9740	0.9848	0.9913
12	39.61	36.43	37.65	37.68	40.31	40.83	0.9906	0.9826	0.9858	0.9880	0.9932	0.9983
13	31.41	29.10	28.62	27.81	29.91	31.94	0.9742	0.9485	0.9473	0.9576	0.9744	0.9835
14	31.81	29.08	32.04	32.01	33.33	32.23	0.9773	0.9561	0.9677	0.9715	0.9821	0.9848
15	35.67	33.81	35.43	36.10	37.51	36.95	0.9834	0.9733	0.9826	0.9842	0.9886	0.9928
16	39.87	37.02	37.20	36.44	41.07	40.43	0.9870	0.9744	0.9767	0.9754	0.9902	0.9937
17	38.56	35.81	36.00	35.53	37.33	38.82	0.9869	0.9745	0.9771	0.9780	0.9862	0.9928
18	33.54	30.74	31.20	31.75	33.50	34.18	0.9756	0.9551	0.9616	0.9678	0.9773	0.9832
19	37.87	34.12	34.35	33.47	38.07	38.40	0.9869	0.9724	0.9747	0.9794	0.9878	0.9932
20	36.74	34.70	36.27	35.48	37.39	38.61	0.9760	0.9761	0.9801	0.9813	0.9866	0.9915
21	35.54	33.22	33.50	33.59	36.85	36.52	0.9846	0.9682	0.9742	0.9782	0.9877	0.9914
22	34.58	32.69	33.44	33.81	35.53	35.65	0.9807	0.9636	0.9706	0.9767	0.9829	0.9884
23	37.67	28.23	36.92	38.56	39.94	38.65	0.9909	0.9759	0.9899	0.9918	0.9935	0.9982
24	32.00	30.84	30.79	29.65	30.91	32.98	0.9734	0.9608	0.9653	0.9642	0.9748	0.9837
Avg.	35.94	33.00	34.41	34.22	36.65	36.81	0.9831	0.9695	0.9758	0.9778	0.9866	0.9910

**Table 2:** Total Average CPSNR(DB) and SSIM of the Kodak and McMaster dataset (Noise-free and with σ= 0.03).

		Noise Free						Noise $\sigma_{n}=0.03$					
**No**.		**CM1**	**CM2**	**CM3**	**CM4**	**CM5**	**PM**	**CM1**	**CM2**	**CM3**	**CM4**	**CM5**	**PM**
Kodak	CPSNR	35.94	33.00	34.41	34.22	36.65	36.81	32.16	31.88	31.88	30.34	32.18	32.32
	SSIM	0.9831	0.9695	0.9758	0.9778	0.9866	0.9910	0.9359	0.9341	0.9385	0.9194	0.9403	0.9391
McM	CPSNR	32.51	30.14	33.14	32.71	33.84	33.17	30.16	28.20	31.03	29.53	30.69	31.28
	SSIM	0.9703	0.9517	0.9748	0.9711	0.9770	0.9733	0.9303	0.9262	0.9430	0.9215	0.9364	0.9321
Kodak + McM	CPSNR	34.47	31.77	33.87	33.57	35.45	35.25	31.30	30.30	31.52	30.00	31.54	31.87
	SSIM	0.9776	0.9619	0.9754	0.9749	0.9825	0.9834	0.9335	0.9307	0.9404	0.9203	0.9386	0.9361

**Table 3:** Average execution time (s) per image.

	CM1	CM2	CM3	CM4	CM5	PM
Time (s)	0.8392	5.5317	110.01	1.3872	1.4478	0.6994

## Data Availability

Not applicable.
